# Effect of Polymeric Matrix Stiffness on Osteogenic Differentiation of Mesenchymal Stem/Progenitor Cells: Concise Review

**DOI:** 10.3390/polym13172950

**Published:** 2021-08-31

**Authors:** Aiah A. El-Rashidy, Sara El Moshy, Israa Ahmed Radwan, Dina Rady, Marwa M. S. Abbass, Christof E. Dörfer, Karim M. Fawzy El-Sayed

**Affiliations:** 1 Biomaterials Department, Faculty of Dentistry, Cairo University, Cairo 11562, Egypt; aiah.abdelwahab@dentistry.cu.edu.eg; 2 Stem Cells and Tissue Engineering Research Group, Faculty of Dentistry, Cairo University, Cairo 11562, Egypt; sarah.mahmoud@dentistry.cu.edu.eg (S.E.M.); esraa.ahmed@dentistry.cu.edu.eg (I.A.R.); dina.radi@dentistry.cu.edu.eg (D.R.); marwa.magdy@dentistry.cu.edu.eg (M.M.S.A.); 3 Oral Biology Department, Faculty of Dentistry, Cairo University, Cairo 11562, Egypt; 4 Clinic for Conservative Dentistry and Periodontology, School of Dental Medicine, Christian Albrechts University, 24105 Kiel, Germany; doerfer@konspar.uni-kiel.de; 5 Oral Medicine and Periodontology Department, Faculty of Dentistry, Cairo University, Cairo 11562, Egypt

**Keywords:** mesenchymal stem cells, polymers, matrix, stiffness, osteoblasts, differentiation

## Abstract

Mesenchymal stem/progenitor cells (MSCs) have a multi-differentiation potential into specialized cell types, with remarkable regenerative and therapeutic results. Several factors could trigger the differentiation of MSCs into specific lineages, among them the biophysical and chemical characteristics of the extracellular matrix (ECM), including its stiffness, composition, topography, and mechanical properties. MSCs can sense and assess the stiffness of extracellular substrates through the process of mechanotransduction. Through this process, the extracellular matrix can govern and direct MSCs’ lineage commitment through complex intracellular pathways. Hence, various biomimetic natural and synthetic polymeric matrices of tunable stiffness were developed and further investigated to mimic the MSCs’ native tissues. Customizing scaffold materials to mimic cells’ natural environment is of utmost importance during the process of tissue engineering. This review aims to highlight the regulatory role of matrix stiffness in directing the osteogenic differentiation of MSCs, addressing how MSCs sense and respond to their ECM, in addition to listing different polymeric biomaterials and methods used to alter their stiffness to dictate MSCs’ differentiation towards the osteogenic lineage.

## 1. Introduction

Stem/progenitor cells are characterized by their outstanding differentiation potential into multiple types of specialized cell lineages, relying on their pluri- or multipotency, while maintaining their self-replicating ability [1,2,3,4,5,6,7,8,9,10]. Among the different stem/progenitor cell types, mesenchymal stem/progenitor cells (MSCs) have been widely used in tissue engineering, cell transplantation, and immunotherapy [11,12,13,14]. MSCs were initially isolated from the bone marrow, but can be currently isolated from almost every tissue in the body [15]. MSCs niches are located in different sites, including umbilical cord blood [16], menses blood [17], dental tissues [18], synovial fluid [19], adipose tissues [14], and dental tissues [4]. MSCs reside adjacent to vessel walls, near perivascular regions, on the endosteal surfaces of trabecular bone, and within the interfibrillar spaces [11].

Proliferation and differentiation of MSCs can be triggered by certain growth factors and chemicals, inducing specific genetic events, affecting the release of transcriptional factors, which regulate the differentiation of MSCs into specific lineages [14,17]. Additionally, biomaterial scaffolds can create a microenvironment that provides MSCs with appropriate conditions for directed differentiation [14]. MSCs further can secrete various immunomodulatory molecules, including cytokines, chemokines, and growth factors, which provide the self-regulated regenerative microenvironment for different injured tissues or organs [13,17].

Regeneration and healing of bone injuries, particularly in large bony defects, is a complicated process [11]. Based on the multipotency of MSCs, they can give rise to either osteoblasts, chondrocytes, myoblasts, or adipocytes in response to key transcriptional regulators that control the primary commitment and most of the follow-up differentiation [20]. MSCs further interact with the components of their local microenvironment (niche) of the extracellular matrix (ECM) [21].

ECM was earlier believed to be an inert matrix that only provides physical support to cells; later, the important role of ECM in various cellular processes was introduced [22]. The MSCs niche provides extrinsic signals including growth factors, ECM, and those released due to contact with other cells. Through these signals, the MSCs’ niche could regulate the stem/progenitor cells’ fate [23,24]. In this context, interactions of MSCs with their niche are reciprocal; thus, MSCs are capable of remodeling the niche in response to signals received from it [24].

Several transcription factors are involved in the osteogenic differentiation pathway, including runt-related transcription factor 2 (Runx2), osterix (Osx, or SP7), Smad, and β-catenin [25,26,27]. Runx2 expressing cells are defined as pre-osteoblasts, a heterogeneous population of cells that includes all cells transitioning from progenitors to mature osteoblasts. A three-stage differentiation of the pre-osteoblasts then follows. The first stage involves cells’ proliferation and expression of transforming growth factor-beta receptor 1 (TGF-βR1), fibronectin, collagen, and osteopontin. The second stage involves the initiation of cellular differentiation and maturation of the ECM with alkaline phosphatase (ALP) and collagen expression. In the final stage, the ECM is enriched with osteocalcin, which promotes matrix mineralization [20]. Runx2 guides MSCs differentiation into osteoblasts besides inhibition of other differentiation pathways, particularly adipogenic and chondrogenic ones [28,29]. Various signaling pathways, such as bone morphogenic proteins (BMPs), Notch, and Wnt signaling pathways, could regulate Runx2 expression.

BMPs are famous for their capability to induce bone formation. They activate intracellular Smad, which translocates to the nucleus and acts as a transcription factor besides promoting Runx2 expression [30]. BMP9 stimulates the activation of Smad1/5/8 in MSCs cells. Moreover, Smad4 knockdown decreases the nuclear translocation of Smad1/5/8 and inhibits osteogenic differentiation [31]. Hence, Smad is of great importance, and its interaction with Runx2 is essential for osteogenic differentiation. Mutation of the C-terminal domain of Runx2 disrupts Runx2–Smad transcriptional activities, which leads to the suppression of osteogenic differentiation [32].

Osx is an essential transcription factor for osteogenic differentiation and subsequent bone formation. In Osx null mice, no bone formation occurred; additionally, in the Runx2 null mice, no expression of Osx was noted, indicating that Osx acts as a downstream of Runx2 and emphasizing its role in MSCs osteogenic differentiation and bone formation [33]. Moreover, Wnt signaling pathway activation in MSCs induces Osx expression and suppresses peroxisome proliferator-activated receptor γ (PPAR-γ) [34]. Moreover, β-catenin has a competitive inhibitory relationship with PPAR-γ, where activation of one of them leads to the degradation of the other [35]. Therefore, Wnt/β-catenin signaling activation shifts MSCs’ commitment towards osteogenesis at the expense of adipogenic differentiation [34].

β-catenin further plays a critical role in MSCs’ osteogenic differentiation. Its absence blocks the osteogenic differentiation and allows for the chondrogenic differentiation of MSCs [36]. Wnt signaling is essential for the β-catenin function. Wnt signaling accumulates β-catenin in the cytoplasm and translocates it into the nucleus, activating the transcription of downstream genes. The absence of Wnt signaling leads to the degradation of β-catenin and interferes with MSCs’ osteogenic differentiation [37]. The sensitivity of β-catenin to matrix stiffness during the differentiation of adipose-derived stromal cells (ASCs) has been demonstrated [38]. β-catenin increased nuclear translocation with increased matrix stiffness and enhanced the expression of Runx2, thus stimulating osteogenesis.

Stem/progenitor cells’ behavior is largely affected by extracellular signals from the microenvironment, including chemical and mechanical cues from the ECM [39]. Unlike chemical cues, the mechanical properties of the microenvironment act as signals that are consistent along with time and space, thus providing long-range stimulation to cells over long periods and over relatively long distances. Recent literature has focused on the paramount role of the ECM mechanical properties in controlling stem/progenitor cells’ behavior, including maintaining their potency, self-renewal and differentiation, migration, proliferation, and interaction with other cells [39,40]. Matrix-related mechanical stimuli, including strain, shear stress, matrix rigidity, and topography, could impact stem/progenitor cell phenotypes through controlling gene transcription and signaling pathways [40,41].

The extracellular-signal-regulated kinase (ERK) and p38 are members of the mitogen-activated protein kinase (MAPK) enzymes family that is concerned with mechanotransduction pathways [42]. ERK is a potent regulator of MSCs’ differentiation, as mechanical stimulation activates ERK through integrin focal adhesion complexes and the initiation of MAPK–ERK signaling cascade [43]. Besides ERK, p38 is involved in MSCs’ differentiation. The p38-MAPK signaling activity has been identified as an essential factor for osteoblastic differentiation [44,45,46]. Ras-mediated signaling has been further presented as a master key that affects multiple intracellular pathways, including ERK, PI3K/AKT, and Smad [47,48]. Inhibition of Ras (RasN17) significantly downregulates AKT, ERK, and Smad1/5/8 activation, as well as osteogenic markers’ expression. Conversely, active Ras (RasV12) has little effect on osteogenic markers’ expression [49]. Consequently, inducing transcription factors to control and guide MSCs’ differentiation has become an essential strategy for guided tissue regeneration [26]. Interference between signaling pathways through interaction between different transcription factors can drive MSCs towards specific cell linage; for example, osteogenic signaling can inhibit the adipogenic signaling pathway, and vice versa [41].

Matrix stiffness has a profound impact on MSCs’ behavior. The adhesion, proliferation, and spreading capacity of umbilical cord MSCs varied when cultured on polyacrylamide gels coated with fibronectin with different stiffness (Young’s modulus: 13–16, 35–38, 48–53, and 62–68 kPa) [50]. Maximum spreading of MSCs was observed with increased matrix stiffness. The soft matrix promoted adipogenic differentiation with high expression of PPARγ and C/EBPα, while MSCs cultured on the 48–53 kPa matrix differentiate into muscle cells with increased expression of MOYG. On the other hand, MSCs cultured on stiff matrix differentiate into osteoblast with increased expression of ALP, collagen type I, Runx2, and osteocalcin [50]. Additionally, bone-marrow MSCs cultured on fibronectin-coated polyacrylamide hydrogels with different stiffnesses, ranging from 13 to 68 kPa, demonstrated enhanced adhesion, spreading and proliferation upon increasing matrix stiffness [51]. On 62–68 kPa, MSCs exhibited a polygonal morphology with a more extensive spreading area and high expression of Runx2, ALP, and osteopontin. These data highlight the critical role of matrix stiffness in regulating MSCs behavior which aids in the development of new biomaterials for tissue regeneration.

Insights into how stem/progenitor cells sense signals from the ECM and how they respond to these signals at the molecular level have become an area of increasing research [21,22,52]. Lately, stem/progenitor cells were shown to be capable of sensing and responding to the structural and functional cues of the matrix [22,52], such as the topography of the ECM components, adhesive properties of the ECM, and ECM stiffness [24,53]. The cells adhere to the ECM via several specific cell-surface receptors, known as integrins [21,22]. Integrins transmit signals from ECM to the cells, thus affecting the proliferation and differentiation of stem/progenitor cells through mechanotransduction of signals [21,22]. It is suggested that the cells use actomyosin filaments (stress fibers) contractility for reciprocal interactions with their matrix [23]. When cells are grown in vitro, extensive efforts to mimic the in vivo microenvironments have been made to control and direct stem/progenitor cell commitment into specific cell lineages required for regenerative medicine.

Natural and synthetic polymeric materials could offer versatile matrices, which are biocompatible and biodegradable, with tunable characteristics, precise control of their topography, and ease of processing [54,55]. Biomaterial stiffness, which determines the material’s resistance to deformation in response to an applied force, is a vital property in tissue engineering. ECM stiffness is calculated by dividing the load by the elastic deformation of the matrix [56], is denoted by the elastic modulus or Young’s modulus (E), and represents the resistance that a cell feels when it deforms the ECM [57].

ECM stiffness guides stem/progenitor cells’ differentiation down corresponding tissue lineages [58]. Osteogenic differentiation of MSCs was shown to be favored on more rigid substrate, while adipogenic differentiation is favored on softer substrates [21]. Such control of MSCs fate by matrix stiffness was shown to be complementary to, and even synergistic with, the regulatory effects of specialized cell culture media commonly used to direct mesenchymal stem/progenitor cell differentiation into specific lineages [23]. Various biomaterials coupled with different methods of controlling stiffness are employed to develop specific stiffness ranges for regulating MSCs differentiation in vitro. Controlling of substrates’ stiffness could be tuned through adjusting the biomaterial composition, the amount/concentration/ratio of material components, the degree of crosslinking, and the reaction conditions [56]. Taking into consideration that the bulk stiffness of most native tissues is much lower than that of plastic or glassware used for in vitro tissue culture [24], the development of biomimetic polymeric matrices of tunable stiffness, mimicking native tissues, allowed new data to reveal more details on the impact of mechanical cues of the microenvironment, especially ECM stiffness, on cellular properties [58].

In this review, we highlight the regulatory role of matrix stiffness in directing the osteogenic differentiation of MSCs, addressing how MSCs sense and respond to their ECM, in addition to listing different polymeric biomaterials commonly used in vitro and methods used to alter their stiffness to dictate MSCs differentiation towards the osteogenic lineage. Moreover, through the current review, we aim to elucidate the effect of ECM stiffness on the MSCs’ osteogenic potential and the underlying mechanism, which is of particular importance during the process of designing new materials for bone-tissue regeneration.

## 2. MSCs and Mechanotransduction

MSCs can sense and assess the stiffness of extracellular substrates [59,60]. The ability of stem/progenitor cells to sense changes in the surrounding environment is known as mechanosensation. Stem/progenitor cells can also transduce mechanical stimuli in the surrounding environment into biochemical signals to induce cellular responses through the process of mechanotransduction [61]. Mechanotransduction is the mechanism underlying the increased osteogenic differentiation of MSCs on stiffer matrix [62]. The process of mechanotransduction in stem/progenitor cells is mediated through focal adhesion, associated integrins, and cellular cytoskeleton, in addition to mechanosensitive ion channels.

### 2.1. Focal Adhesion and Integrins

**Figure 1:**
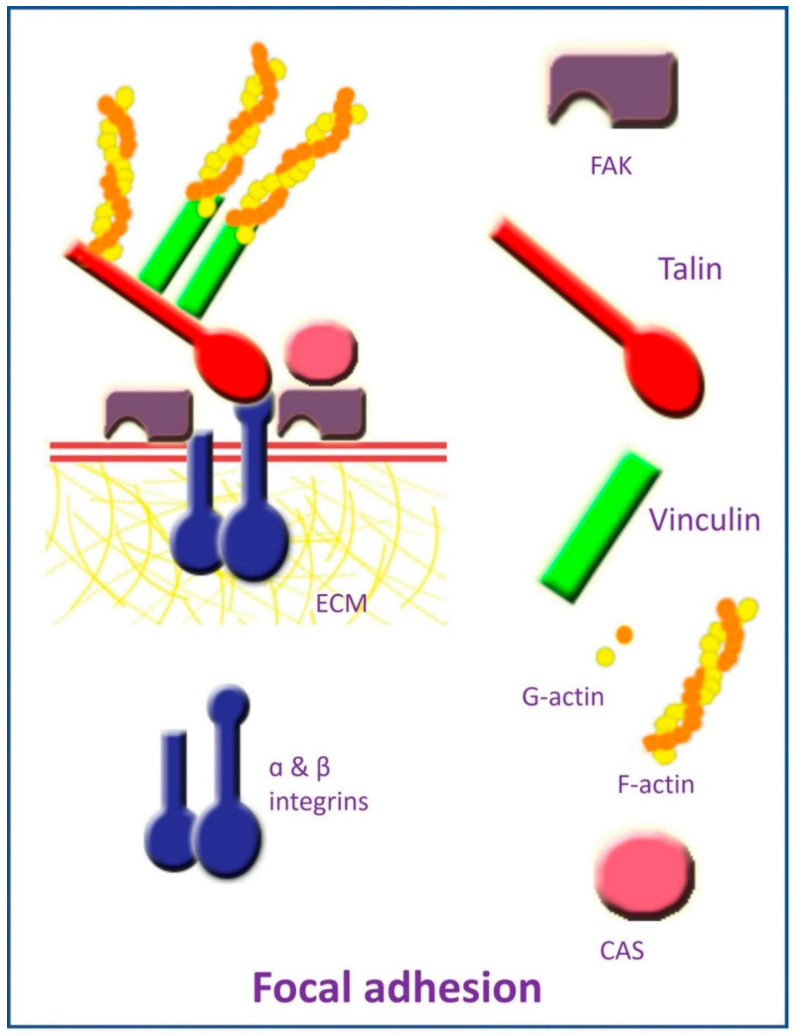
Elements of focal adhesion.

Focal adhesions are complexes of highly specialized proteins and macromolecules that can attach cells to the ECM, allowing them to sense and respond to mechanical stimuli [63]. Focal adhesion is composed of the transmembrane protein integrin, which has intracellular and extracellular domains (Figure 1). Integrin’s intracellular domain is linked to the actin cytoskeleton via cytoplasmic adapter proteins, which include the actin-binding proteins α-actinin, vinculin, and talin [64]. The integrin extracellular domain binds to ECM components, such as collagen, laminin, and fibronectin, via its extracellular domain, thereby establishing a mechanical connection between ECM and intracellular cytoskeleton components [64,65,66]. Fifty different proteins have been associated with focal adhesion [67], including intracellular proteins as focal adhesion kinase (FAK) and p130Cas [68,69].

Integrins are alpha and beta subunits heterodimers existing in different combinations [64]. There are 18α and 8β subunits, which account for 24 different integrin heterodimers in mammals specific to an exact set of ECM ligands [70,71]. Through their intracellular and extracellular domains, integrins are capable of joining intracellular cytoskeleton with the external environment, thereby creating mechanical integration between ECM and intracellular cytoskeleton [72]. They can transmit cellular signals to the ECM, and reciprocally can convey signals from the ECM intracellularly [73], triggering an intracellular signaling pathway, resulting in alteration of cellular migration, proliferation, and differentiation [74].

It is noteworthy that MSCs’ surface integrins’ expression can influence MSCs’ lineage commitment [75]. Further, matrix stiffness can influence integrin expression on MSCs, which can dictate and direct stem/progenitor cell fate [76]. Undifferentiated MSCs were found to mostly express α1, α3, αV, β1, and β2 integrins, while α2, α4, α5, α6, β3, β4, and β5 were expressed to a lesser extent [77]. MSCs’ osteogenic differentiation was reported to be associated with upregulation of integrin α5 expression on MSCs’ surface in response to ECM stimuli [77,78,79,80]. Integrin α5 upregulation promotes osteogenesis through activation of FAK via the ERK1/2-MAPKs and PI3K signaling [79]. MSCs’ expression of integrin subunits α2 [62,76], α1, αV, and β3 was also upregulated with increased ECM stiffness, favoring osteogenic differentiation [62], while α5 and β1 expression was upregulated in the matrix with lower stiffness [76]. Additionally, activation of MSCs expression of α5β1 and αVβ3 integrin complexes in response to ECM morphology was associated with enhanced osteogenic differentiation [81]. On the other hand, osteogenic differentiation was associated with reduced expression of α1, α3, α4, β3, and β4 integrin subunits [77], while MSCs’ adipogenic differentiation was associated with the upregulation of α6 and reduction of α2, α4, α3, β3, and β4 integrin subunits expression [77]. Increased integrin α5 expression can also inhibit both adipogenic and chondrogenic differentiation, while promoting MSCs osteogenic differentiation [82].

The binding of integrin to ECM components triggers the intracytoplasmic assembly of focal adhesion proteins, including talin, FAK, p130Cas, and vinculin, first forming focal complexes, which then grow, giving rise to focal adhesions, linking actin fibers to ECM components [83]. Formation of focal adhesion, with the associated triggering of intracellular signaling pathways, is essential for MSCs migration, proliferation and differentiation [84,85,86].

MSCs lineage commitment and osteogenic differentiation in response to ECM mechanical cues, including matrix stiffness, involve upregulation of focal adhesion formation. Increasing matrix stiffness can promote number [84] and area of focal adhesions [87]. In turn, upregulation of focal adhesion number and size [60,88,89] has been linked to increased osteogenic differentiation of MSCs. Additionally, tightly packed focal adhesion can stimulate osteogenic differentiation [90].

### 2.2. Cytoskeleton Elements 

Cytoskeletal-related proteins are responsible for the ability of stem/progenitor cells to respond to mechanical cues, including stiffness of the ECM [85]. In addition to their role in providing a cellular structural framework, repolarization of cytoskeleton elements in response to mechanical stimuli, transmits the signals from the ECM to the nucleus, resulting eventually in altered gene expression [64,91,92]. Structural elements of the cellular cytoskeleton include microfilaments, intermediate filaments, and microtubules [64].

**Figure 2:**
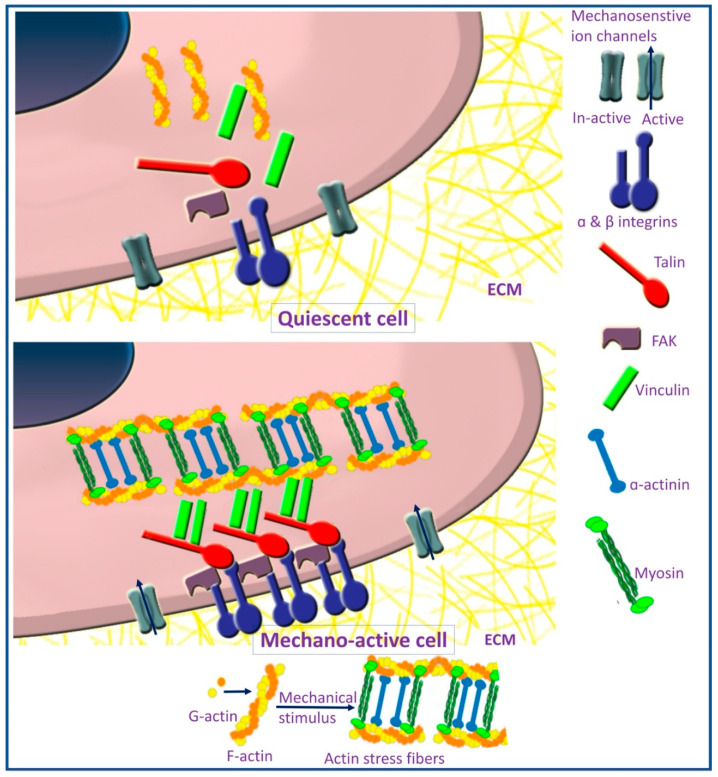
Focal adhesion formation and stress fibers assembly in mechano-active cells.

The actin cytoskeleton is responsible for the maintenance of cell shape, motility, and contractility. They also act as mechanical sensors for the extracellular environment [93]. It is formed of F-actin, which is a helical polymer of G-actin coupled with actin-binding and actin-bundling proteins, such as α-actinin, vinculin, and talin [94] (Figure 2). Actin cytoskeleton forms a web in association with cellular junctions and forms a core of microvilli, filopodia, and lamellipodia [64]. Actin perinuclear cap is a dome-like structure formed of contractile actin filament and phosphorylated myosin, covering the top of the nucleus and connected to the nucleus through linkers of nucleoskeleton and cytoskeleton (LINC) protein complexes [95,96]. This actin perinuclear cap provides a mechanism through which mechanical signals, transduced through focal adhesion, can reach the nucleus to induce cellular responses [95,97]. Ultimately, the actin perinuclear cap is responsible for conveying signals regarding matrix stiffness to the nucleus [97].

The binding of MSCs to a stiff substrate induces actin polymerization, as evident by an increased ratio of F-actin to G-actin, forming actin stress fibers, which trigger intracellular-signaling pathways [67]. Stress fibers are actomyosin complex composed of F-actin and myosin-2 stabilized by crosslinking proteins [98] (Figure 2). The process of actin polymerization is regulated by the FAK signaling pathway [99]. Actin polymerization and stress fibers formation are essential for establishing cell to ECM interaction [100]. Polymerization of actin dictates lineage commitment of MSCs, as actin depolymerization was noticed during adipogenic differentiation [101]. On the contrary, actin polymerization combined with an increased ratio of F-actin to G-actin upregulated osteogenic differentiation [99,102,103,104,105]. On the other hand, disruption of actin polymerization can reduce osteogenic differentiation [103]. Increased osteogenic differentiation on stiffer substrates was also associated with increased expression of F-actin [78], in addition to actin-binding protein (vinculin) [106].

Actin filaments can also interact with other components of the cellular cytoskeleton as intermediate filaments [64]. Intermediate filaments have a diameter of about 10 nm and have a role in maintaining cell shape and cellular junctions [64]. F-actin promotes intermediate filaments and vinculin assembly and disassembly, which are required for the process of osteogenesis through the transient receptor potential melastatin 7–osterix axis [107].

### 2.3. Mechanosensitive Ion Channels

Mechanosensitive ion channels are a further mechanism implicated in MSCs’ mechanotransduction on stiff matrices. These ion channels are sensitive to substrate stiffness. Upon mechanical stimulation, they allow the intracellular influx of ions and can form complexes with stress fibers, eliciting intracellular signaling pathways [108]. Mechanical stimulation affects cell differentiation through a change in calcium influx through activated channels [109]. The change in the calcium influx results in the activation of the MAPK signaling pathway [110].

### 2.4. MSCs’ Aging and Mechanosensitivity

Different age-dependent changes in MSCs were reported, such as decreased proliferation ability [111] and osteogenic differentiation potential [112,113,114]. Moreover, age-associated bone loss was linked to the reduced osteogenic potential of MSCs [115]. Aged multipotent progenitor cells lose their sensitivity to alterations in polyacrylamide substrates, while younger multipotent progenitor cells showed a lineage-dependent response to stiffness [116]. The effect of MSCs aging on their mechanosensitivity was investigated by comparing the response of child (11 to 12 years old) and adult MSCs (20–30 years old) to variations in stiffness (10 and 300 kPa) of type I collagen-coated polyacrylamide substrates [117]. Child MSCs revealed more mechanosensitive (increased nuclear-translocation of YAP), improved angiogenesis (enhanced endothelial tubule formation), and osteogenesis (increased alkaline phosphatase activity and mineralization) on stiff substrates as compared to adult MSCs. Based on a customized PCR array, an age-dependent, stiffness-induced upregulation of NOX1, VEGFR1, VEGFR2, WIF1, and JNK3 in child MSCs compared to adults MSCs [117]. Understanding the mechanism behind the age-altered mechanosensitivity of MSCs may open up new avenues to identify potential therapeutic targets to reproduce the enhanced osteogenic and angiogenic potential of adults with bone degeneration and disease.

## 3. The Role of Matrix Stiffness in Triggering MSCs’ Osteogenic Differentiation 

**Figure 3:**
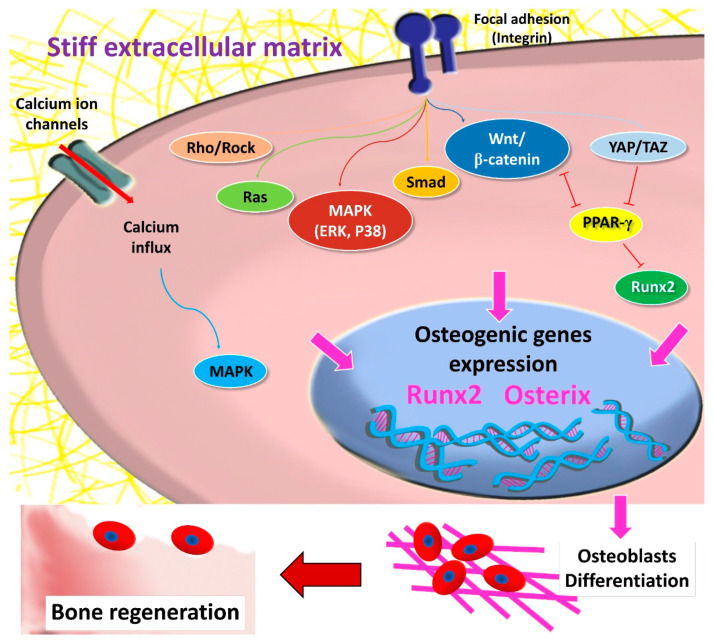
Signaling pathways involved in stiffness induced MSCs’ osteogenic differentiation.

Matrix stiffness regulates the MSCs’ differentiation into mature specific cells by activating transcription factors that upregulate genes responsible for the initiation and progression of particular cell-linage differentiation. The singling pathways involved in MSCs’ osteogenic differentiation are illustrated in (Figure 3). Rigid matrices led to increased MSCs spreading and improved actomyosin contractility, promoting osteogenic differentiation. This enhanced potential was accompanied by increased Runx2, β-catenin, and Smad, implying the significant impact of mechanosensing the matrix stiffness and its role in determining the cell fate [41,50]. The relation between Runx2 expression, owing to mechanosensation with actomyosin contractility, was confirmed by inhibiting myosin, which caused a decrease in Runx2 expression [118]. However, the effect of matrix stiffness on MSCs’ differentiation disappeared at the monolayer state [49].

The hippo pathway is one of the signaling pathways involved in MSCs’ differentiation and is regulated by intra- and extracellular signals [119]. The downstream effectors of the hippo signaling pathway are yes-associated protein (YAP) and transcriptional co-activator with PDZ-binding motif (TAZ) [120]. YAP and TAZ transduce signals necessary for determining MSCs’ fate. The control of the Hippo pathway is through phosphorylation and nuclear translocation of YAP/TAZ [121]. Additionally, matrix stiffness can control the localization and activity of YAP/TAZ [122,123], which is identified through the structural and functional regulation of the cell cytoskeleton to adjust cellular tension [124]. The stresses sensed by MSCs are transmitted to the nucleus and lead to an increase in the nuclear membrane tension, causing expansion of the nuclear pores, which promote sudden nuclear inflow of YAP [125]. In MSCs cultured on a rigid matrix (40 kPa) undergoing osteogenic differentiation, YAP/TAZ has been localized in the nucleus. In comparison, MSCs cultured on a soft matrix (0.7 kPa), YAP/TAZ persisted in the cytoplasm, directing MSCs to undergo adipogenic differentiation [122]. Moreover, YAP knocking-down resulted in inhibition of osteogenesis and enhancement of adipogenesis [126]. During MSCs’ osteogenic differentiation, TAZ functions as a co-activator of Runx2 to stimulate osteogenesis and inhibits PPAR-γ, which reduces adipogenic differentiation [127]. These findings highlight the significant role of YAP/TAZ as a potent regulator of stiffness-induced osteogenic differentiation.

MSCs fate is also directed through actomyosin contractility and activated Rho/Rho kinase (ROCK) signaling [88], along with mechanotransduction mediated by focal adhesion and integrin [128]. In response to increased stiffness, activated Rho stimulates actomyosin stress fiber assembly [129], which causes an increase in cell contractility and activation of ERK, promoting osteogenic differentiation [130]. Furthermore, Rho combined with the actin cytoskeleton is essential to maintain nuclear YAP/TAZ in MSCs [122]. Activation of FAK via ROCK signaling led to upregulation of osteogenic marker Runx2, ALP, and matrix mineralization denoting osteogenesis of human adipose stem/progenitor cells [131]. In addition, the inhibition of FAK and ROCK signaling caused an upregulation of adipogenic markers. Furthermore, matrix stiffness modulates MSCs’ osteogenic differentiation through the Ras pathway, which is accompanied by an increase in the phosphorylation levels of Smad1/5/8, AKT and ERK [49]. Ras (RasN17) inhibition resulted in a significant decrease of Smad1/5/8, AKT, and ERK activity, as well as osteogenic markers’ expression [49].

Cells on stiff matrices develop high cytoskeletal tension, which is evidenced by enhanced actin stress fibers and large spread area. Below a compressive modulus of 25 kPa, regardless of the adhesive ligand presented, there is not enough cytoskeletal tension to promote osteogenic lineage differentiation [88]. Based on these results, it has been postulated that, unless a cell develops cytoskeletal tension exceeding a certain threshold stiffness (substrates with moduli of ≥ 25 kPa), osteogenic differentiation will not occur and the cell would need the presence of an osteogenic ligand for Runx2 expression for further differentiation to take place. On the other hand, MyoD1 (a marker for myoblasts) expression demonstrated less ECM dependence compared with Runx2, as it was markedly expressed in cells cultivated on substrates with stiffnesses higher than 9 kPa, regardless of the protein coating [132]. Additionally, on soft poly(acrylamide-co-acrylic acid) substrates (E = 15.4 kPa) that mimic muscle elasticity when grafted with arginine–glycine–aspartate (RGD) peptide sequence, MSCs were directed to a spindle-shaped morphology similar to C2C12 myoblasts, while stiffer matrices (E = 47.5 kPa) that mimic osteoid tissue’s crosslinked collagen yield the cells in polygonal morphology, similar to MC3T3-E1 pre-osteoblasts [118].

Inflammation can further counteract the inductive effect of matrix stiffness on osteogenic differentiation. Periodontal ligament stem cells (PDLSCs) cultured with the inflammatory cytokine interleukin (IL)-1β on gelatin/methacrylate hydrogels with different matrix stiffness showed a marked reduction in matrix stiffness-dependent osteogenic differentiation and expression of osteocalcin, as well as Runx2. This was through the activation of p38 signaling pathways, which were activated by IL-1β [133]. Further, macrophages encapsulated in gelatin/methacrylate hydrogels with high stiffness showed a high tendency to polarize toward the pro-inflammatory M1 phenotype, which was associated with a negative impact on the osteogenic differentiation of bone-marrow mesenchymal stem cells (BMMSCs) [134].

## 4. Matrix-Dependent MSCs’ Osteogenic Differentiation 

Several natural and synthetic polymeric biomaterials are currently used in tissue engineering and regenerative medicine, serving as biomimetic matrices for in vitro culturing [135]. These biopolymers can be generally divided into two classes: natural and synthetic polymers. Natural polymers include alginate, collagen, gelatin, hyaluronic acid, elastin, actin, keratin, albumin, chitosan, and others. They are characterized by their inherent bioactivity and ability to mimic natural tissues, yet they suffer from possible immunogenicity, structural complexity, and poor mechanical properties. Chitosan can be used for increasing energy storage of α-cobalt molybdate (CoMoO) nano-flakes in the presence of a crosslinking agent such as citric acid [136]. Compared to natural polymers, synthetic polymers have higher mechanical properties, are readily available, with tunable physicochemical properties and degradation rate, but lack natural tissue resemblance [55,137]. Major synthetic polymers used include polyethylene glycol (PEG), polydimethylsiloxane (PDMS), polyesters, polyacrylamide, vinyl polymers, and self-assembling peptides, in addition to poly (lactic acid) (PLA), poly (glycolic acid) (PGA), poly (lactide-co-glycolic acid) (PLGA), and others [55,135].

### 4.1. Natural Polymers

#### 4.1.1. Alginate

Alginates extracted from seaweeds and algae are composed of β-1,4-linked blocks of β-D-mannuronic acid (M) and its C-5 epimer α-L-guluronic acid [138]. Alginates are widely used polysaccharides for hydrogelation in tissue engineering as they can be gelated easily through the addition of divalent cations [139]. Since alginate could act as a template for binding of manganese ions, the presence of a high concentration of alginate in the electrolytic manganese dioxide altered the morphology from spindle-shaped to cactus-shaped [140].

Spatially modulating the mechanical properties in an alginate bioink, 3D printed constructs were postulated to regulate MSCs’ fate. Micro-CT-based imaging with 3D bioprinting and bioreactor system were utilized to fabricate 3D human MSCs-laden porous bone-like scaffolds with varying compressive moduli based on two unmodified polymers (alginate and gelatin). Softer scaffolds with low alginate concentration (0.8% alginate, 0.66 ± 0.08 kPa) revealed accelerated and enhanced osteogenic differentiation with upregulated ALP activity than stiffer scaffolds (1.8% alginate, 5.4 ± 1.2 kPa). In the presence of the osteogenic differentiation medium, cells on soft scaffolds exhibited osteoblastic and early osteocyte-related gene expression and showed a 3D cellular network within the mineralized matrix [141].

Increasing alginate molecular weight, as well as increasing the crosslinking ratio, produces a significantly stiffer bioink. Upon bioprinting cylindrical MSCs laden constructs with spatially variable mechanical stiffness from the core to the periphery, more MSCs underwent osteogenic differentiation within the stiffer regions of the printed constructs as evident by increased ALP staining [142]. In contrast to most studies, an investigation demonstrated that, under basal conditions and in the absence of RGD ligands, alginate hydrogel with bimodal molecular weight distribution (50% LMW and 50% HMW) and 1 wt.% polymer concentration of low-stiffness 3D matrices (tan ∂ ≈ 0.4–0.6) provided a permissive environment for human MSCs osteogenic differentiation and expressed high levels of ALP and osteocalcin as compared to the stiffer 2 wt.% alginate hydrogel with the presence of RGD ligands [143].

#### 4.1.2. Collagen

Collagen is considered to be the most abundant protein in mammals [144]. Being the main ECM protein, collagen, together with soluble factors, may act as a niche for MSCs osteogenesis and bone mineralization [145]. Mechanical properties of collagen fibers vary depending upon their location in different tissues. Thus, the cells can sense local fibrillar microenvironments with different physical cues. Collagen gels were engineered to attain varying fiber stiffness (from 1.1 to 9.3 kPa), while maintaining bulk stiffness below 200 Pa, by changing the polymerization temperature to 4 °C (Col-4), 21 °C (Col-21), and 37 °C (Col-37) without changing the density of the collagen. A polymerization temperature of 4 °C led to shorter, thicker, and stiffer collagen fibers (Col-4), with limited fiber recruitment and force transmission and fewer focal adhesions. Cells grown on Col-4 showed much slower spreading as compared to Col-37 with similar bulk stiffness but with more flexible and longer fibers that can be easily remodeled. Human MSCs cultured on Col-4 revealed a much lower ratio of osteogenic differentiation (21.1%) compared to that on Col-37 with (34.1%) ALP positive reactivity [146].

Matrix stiffness possesses a high impact on cellular bioactivity regardless of the presence or absence of growth factors. This was proved by culturing porcine adipose-derived stem cells (ADSCs) on sequentially integrated benzophenone photo-immobilization and carbodiimide, crosslinking collagen–glycosaminoglycan, with stiffness ranging from 2.85 to 5 MPa, in the presence or absence of covalently immobilized platelet-derived growth factor (PDGF-BB) and BMP-2 [147].

In the presence of osteogenic culture media, human MSCs coated with three bilayers of collagen/alginate nanofilm with relatively high stiffness 24 and 53 MPa revealed an augmented osteogenic differentiation efficiency with a significant increase in ALP by activating transcriptional co-activators with the PDZ binding motif through extracellular signal-related kinase and p38-MAPK signaling [148].

Nanoparticulate mineralized collagen–glycosaminoglycan scaffolds chemically crosslinked with 1-ethyl-3-(3-dimethylaminopropyl) carbodiimide and N-hydroxysuccinimide had a higher range of elastic moduli 3.90 ± 0.36 kPa as compared to non-crosslinked materials. Cultured human MSCs on crosslinked substrates showed higher expression of osteogenic genes and proteins compared to non-crosslinked versions. This was maintained via the mechanotransduction mediators YAP/TAZ and the Wnt signaling pathway [149].

MSCs seeded within a 3D collagen gel with an elastic modulus of ~108 Pa stimulated by vibrations of nanoscale amplitude in a vibrational bioreactor showed increased expression of Runx2, collagen I, ALP, osteopontin, osteocalcin, and BMP-2. This indicated that the viscoelastic properties of the collagen gel allowed the transfer of high-frequency vibrations to the cells seeded in 3D [150].

In the absence of any differentiation supplementation, MSCs grew on stiffer (1.5 kPa) dehydrothermal and 1-ethyl-3-3-dimethyl aminopropyl carbodiimide (EDC) crosslinked collagen–glycosaminoglycan scaffolds showed the greatest level of Runx2 expression, while substrates with lower stiffness (0.5 kPa) resulted in significant elevation of SOX9 expression, indicating that MSCs are directed towards a chondrogenic lineage [151].

Additionally, 3D scaffolds with the highest proportion of collagen in collagen and hydroxyapatite mixture coated on the decellularized cancellous bone with various stiffness (13.00 ± 5.55 kPa, 13.87 ± 1.51 kPa, and 37.7 ± 19.6 kPa) exhibited the highest stiffness that, in turn, promoted higher expressions of osteopontin and osteocalcin [152].

#### 4.1.3. Gelatin

Gelatin is a natural, biocompatible, non-immunogenic, hydrophilic, and biodegradable collagen derivative [153,154,155]. It is acquired via acid or alkaline hydrolysis of collagen into single molecules [156]. Being derived from a natural source, gelatin is characterized by having RGD cell-binding motifs that can enhance cellular attachment [157]; it can also promote cell proliferation and differentiation [158,159]. One of the major disadvantages of gelatin is its low mechanical properties [153,160], in addition to its thermal instability [161].

Gelatin crosslinking through the addition of chemical groups can help reduce these shortcomings [161]. Stiffness of gelatin hydrogel scaffold can be controlled through changing the ratio of crosslinking agents, such as methacryloyl, giving rise to gelatin–methacrylate (GelMA) [133,162,163], transglutaminase [134], or EDC [164], and through the incorporation of variable additives as starch [162] or polyethylene glycol diacrylate (PEGDA) [163]. Increased gelatin hydrogel scaffold degree of crosslinking and matrix stiffness were positively associated with increased osteogenic differentiation of MSCs [133,134,162,163,164]. Crosslinked gelatin hydrogel can also be modified to enhance osteogenic potential through incorporation of the bisphosphonate alendronate [163]. GelMA hydrogel with tunable stiffness was constructed by using different GelMA concentrations 10, 12, and 14 wt to yield hydrogel with stiffness 25.75 ± 1.21 kPa, 59.71 ± 8.87 kPa, and 117.82 ± 9.83 kPa, respectively. Osteogenic differentiation of PDLSCs, as well as expression of osteocalcin and Runx2, showed a significant increase with increasing matrix stiffness through activation of ERK1/2 signaling pathway [133].

Gelatin/starch-based hydrogel was fabricated with tunable stiffness from crosslinked gelatin with variable degrees of methacrylation (GelMA; 31%, 72%, and 95%) covalently bound to variable ratios of pentenoates modified starch (10 v% starch and 20 v% starch). Increasing the degree of methacrylation and combining crosslinked gelatin with starch, with subsequent increase in matrix stiffness, effectively promoted osteogenic differentiation of adipose stem cells (ASCs), as was evident by an increased ALP expression. GelMA 95% combined with starch showed the highest degree of osteogenic differentiation, while the highest degree of adipogenic differentiation was observed on the least crosslinked and most flexible gelatin hydrogel (GelMA 31%) [162].

Three-dimensional porous gelatin scaffolds crosslinked using EDC further demonstrated an increase in the elastic modulus from ~0.6 to ~2.5 kPa, without any change in the scaffold internal structure. BMMSCs cultured on EDC-crosslinked gelatin scaffolds with increased stiffness showed an increased osteogenic differentiation as evidenced by increased Runx2 and osteocalcin expression in vitro. Subcutaneous implantation of EDC-crosslinked gelatin scaffold loaded with BMMSCs transfected with adenovirus encoding BMP-2 in mice demonstrated an increased bone formation in vivo, as compared to the control, non-crosslinked scaffold with low stiffness [164].

Transglutaminase-crosslinked gelatin scaffold with variable stiffness was constructed, using gelatin concentrations of 3%, 6%, and 9%. The 9% gelatin gave rise to the highest stiffness (60.54 ± 10.45 kPa), while 3% gelatin resulted in the lowest stiffness (1.58 ± 0.42 kPa). BMMSCs encapsulated in the hydrogel with the highest stiffness demonstrated the highest osteogenic differentiation as revealed by ALP activity, calcified nodule formation, expression of SP7 transcription factor-2, Osx, Runx2, and osteocalcin [134].

Augmentation of GelMA with alendronate and PEGDA showed a positive effect on osteogenic differentiation of BMMSCs. GelMA and alendronate were added at different concentrations and grafted on gelatin molecules, followed by further crosslinking, using 20 wt.% PEGDA to improve hydrogel scaffold stiffness from 4 to 40 kPa. Osteogenic differentiation of grafted BMMSCs was promoted on stiffer hydrogel with higher alendronate concentration, as evident by upregulation of ALP activity, collagen type I, and osteocalcin expression, as well as calcium deposition [163].

#### 4.1.4. Decellularized Matrix and Demineralized Bone

Decellularized cell-derived matrices (dCDMs) could further provide a way to mimic natural tissues. It has been reported that aligned dCDMs could contribute to producing a more homogeneous environment, which resulted in a uniform response of cells to the biophysical environment, displaying a highly homogeneous phenotype and can undergo differentiation if properly stimulated [165]. Substrates displaying linear topographic patterns were obtained by replica molding, using PDMS, while flat PDMS substrates were produced by using a polystyrene dish as a control.

Cell-derived matrices (CDMs) were attained by cultivating MC3T3-E1 pre-osteoblasts in the presence of ascorbic acid for two weeks on linear or flat surfaces. MC3T3-E1 cultivated on nanopatterned substrates produced an aligned fibrillar matrix, whose microarchitectural features remained intact after the decellularization process. Atomic force microscope measurements performed on bare dCDMs revealed very low Young’s moduli in the range of (0.01–0.1 kPa) that was increased by genipin crosslinking to reach (i.e., 0.1–1.5 kPa). These matrices were further seeded with murine MSCs and cultivated in the presence of either osteogenic or adipogenic media for two weeks. Both the aligned and random dCDMs promoted murine MSC adhesion and proliferation in their pristine state, while maintaining high levels of stemness markers, with a more homogeneous population of undifferentiated cells, were seen on aligned dCDMs. On the pristine dCDMs, MSCs promptly underwent adipogenic differentiation when stimulated with induction media, while they were minimized in the presence of osteogenic medium, due to very low stiffness. On the contrary, MSCs responded consistently on stiff dCDMs, displaying a significant adipogenic and osteogenic differentiation potential [165].

In another study, 3D demineralized bone matrices with the same 3D microstructure (porosity and pore size), but with various compressive moduli (high: 66.06 ± 27.83 MPa, medium: 26.90 ± 13.16 MPa, and low: 0.67 ± 0.14 MPa), were fabricated by controlling the decalcification duration (1 h, 12 h, and 5 d, respectively). Low-stiffness scaffolds promoted BMMSCs’ osteogenic differentiation. Subcutaneous implantation in a rat model further revealed efficient improvement of cells’ infiltration and deposition of collagen fibers, in addition to upregulated positive osteocalcin and osteopontin expression, as well as angiogenesis upon utilizing the low-stiffness scaffolds. Further implantation in a femoral condylar defect rabbit model supported the previous findings and revealed that stromal-cell-derived factor-1α/CXC chemokine receptor (SDF-1α/CXCR4) signaling pathway was essential for the stiffness-mediated stem/progenitor recruitment and osteogenic differentiation during bone repair [166].

#### 4.1.5. Hyaluronic Acid

Hyaluronic acid (HA) is a linear non-sulfated polysaccharide made up of repeated disaccharide molecules in alternating patterns (D-glucuronic acid and N-acetyl-D-glucosamine). This pattern is linked through interchanging β-1,4 and β-1,3 glycosidic bonds. HA is a fundamental component of ECM that regulates various cellular biological processes, such as migration, adhesion, proliferation, and differentiation, through binding with a specific receptor on the target cell [167,168]. Owing to its exceptional biocompatible, biodegradable and non-immunogenic properties, HA is clinically used for drug delivery and tissue regeneration [9,169,170]. As being a natural extracellular component, HA mimics the typical ECM and could initiate signaling pathways responsible for osteogenesis [171,172]. Moreover, the physicochemical and biological properties of HA could be altered by chemical modification [173].

Through adjusting the crosslinker (PEGTA) density, a series of hydrogels with different biochemical and biomechanical properties were developed by utilizing a thiol-functionalized HA and a thiol-functionalized recombinant human gelatin. Human BMMSCs were cultured on the hydrogels with different stiffness (storage modulus (G′) and corresponding PEGTA concentrations, namely 0.15 kPa (0.25%), 1.5 kPa (1.75%), and 4 kPa (2.5%), in adipogenic and osteogenic conditions. Adipogenic differentiation was confirmed by gene expression of lipoprotein lipase (LPL), as well as PPARγ2, with similar LPL expression levels demonstrated on the hydrogels with varying stiffness, whereas PPARγ2 expression was markedly enhanced upon increasing hydrogel stiffness. Cells exhibited spindle-shape morphology on the 0.15 kPa hydrogel, while displaying elongated and cuboidal appearance, similar to osteoblasts on greater stiffness hydrogels. Human MSCs cultured on the 1.5 kPa hydrogel significantly expressed osteopontin, while those cultured on the 4 kPa hydrogel revealed a significant upregulation in the expression of the late osteogenic gene (bone sialoprotein) [174].

In a novel HA hydrogel platform, ligation of the HAVDI adhesive peptide sequence from the extracellular N-cadherin domain 1 and the RGD adhesive motif from fibronectin led to Rac1-GTP-dependent reductions in the attachment of myosin IIA to the focal adhesions. This lack of myosin IIA incorporation into focal adhesions hindered the maturation of these adhesions with increasing substrate stiffness (E = 5, 10, and 15 kPa) and thereby decreased traction force generation on the underlying substrate. These alterations in the mechanical state of the MSCs further reduced mechanosensitive YAP/TAZ translocation to the nucleus, herewith attenuating the signaling pathways involved in mesenchymal development, including cell proliferation and osteogenic differentiation [175].

An in vitro culture system for osteochondral tissue engineering was developed, using HA gels with various stiffness (G′ ranging from 10 to 45 Pa) attained by mixing Glycosil^®^, a thiol-modified hyaluronan gel with the crosslinking agent PEG at ratios from (1:1 to 7:1). The co-differentiation media (a ratio of 50% chondrogenic:50% osteogenic) proved to be suitable for appropriate chondrogenic and osteogenic differentiation of human MSCs. On the stiffest matrix (HA:PEG construct at a 2:1 ratio), the three chondrogenic markers (aggrecan, collagen II, and sox 9) were expressed by the differentiated human MSCs cultured for 21 days [176].

Moreover, human BMMSCs were initially entrapped in a HA carrying sulfhydryl groups and a hydrophilic polymer bearing both acrylate and tetrazine groups with the shear elastic modulus (G′) =180 ± 42 Pa. The stiffness of the matrix was increased (G′ = 520 ± 80 Pa) by adding HA conjugated with multiple copies of trans-cyclooctene (TCO) to the human MSCs-laden gel culture media. The 3D matrix tagged with a TCO-cell-adhesive motif promoted the cells to undergo remarkable actin polymerization, changing from a rounded phenotype to a spindle morphology with long processes. After an additional seven days of culture in the modified media, quantitative analysis showed that RGD tagging enhanced cellular expression of matrix metalloproteinase 1, whereas it decreased the expression of tenascin C and collagen I/III. RGD tagging, however, was not sufficient alone to induce chondrogenic, adipogenic, fibroblastic/myofibroblastic, or osteogenic differentiation [177].

Photo-crosslinked methacrylated HA hydrogels incorporating fragmented polycaprolactone (PCL) nanofibers with compression modulus 3122.5 ± 43.7 Pa promoted osteogenic differentiation of adipose-derived stem/progenitor cells incorporated into the composite hydrogel. The biomarkers collage type 1, ALP, and Runx2 were significantly expressed in the hydrogels containing nanofibers. In addition, the results of alizarin red staining confirmed osteogenic differentiation [178].

#### 4.1.6. Fibrin

Fibrin is one of the natural biopolymers that offers many advantages based on its excellent biocompatibility and cell-adhesion properties [179]. However, fibrin has low mechanical properties that can be modified by adjusting the concentration and ionic strength of fibrinogen to obtain a polymeric substrate mimicking native ECM [55]. A high concentration of fibrinogen and thrombin resulted in a stiffer fibrin matrix, as compared to fibrin with lower fibrinogen and thrombin concentration, as altering these two components allowed the tuning of fibrin elasticity. Microfluidic biochips coated with stiff fibrin substrates modified with gold-nanowires-enhanced osteogenic differentiation of human amniotic mesenchymal stem cells (AMSCs) and led to significant elevation in collagen type I levels and matrix mineralization (calcium deposition), while softer fibrin matrices with lower fibrinogen and thrombin concentration enhanced human AMSCs chondrogenic differentiation [180].

### 4.2. Synthetic Polymers

#### 4.2.1. Polyethylene Glycol

Polyethylene glycol (PEG) is one of the most widely used synthetic polymers in the tissue engineering field. It is characterized by being chemically and biologically inert and by the high hydrophilicity of the polymer backbone. There is a wide range of polymer architectures and lengths that are commercially or synthetically accessible [181].

MSCs cultured on 3D thixotropic PEG-silica nanocomposite gel with high stiffness (≥75 Pa) expressed the highest level of Runx2 and osteocalcin. Additionally, RGD cell-adhesion peptide sequence immobilization in the gel of 75 Pa stiffness promoted ~13% higher expression of the osteogenic transcription factor [182]. Rat BMMSCs cultured on relatively soft (130 kPa) and stiff (3170 kPa) PEG hydrogels with RGD nano-spacings of 49 and 135 nm, incubated in the mixed osteogenic and adipogenic medium, exhibited a higher density of adherent MSCs, and osteogenesis was promoted on stiffer hydrogels. When the hydrogel stiffness was controlled, the large RGD nano-spacing was beneficial for osteogenesis, while the small RGD nano-spacing generated more adipogenesis [183].

Human MSCs were encapsulated in a multilayer PEG-based hydrogel composed of a soft cartilage-like layer of chondroitin sulfate (48 kPa) and low RGD concentrations, a stiff bone-like layer 345 kPa with high RGD concentrations, and an intermediate interfacial layer with 100 kPa. The recorded stiffness of the multilayer hydrogel was 90 kPa. Opposite to static conditions, dynamic mechanical stimulation generated a high expression of collagens with collagen II in the cartilage-like layer, collagen X in the interfacial layer, and collagen I in the bone-like layer with the presence of mineral deposits in the bone layer [184].

PEG/silk fibroin/HA (PEG/SF/HA) scaffold was prepared with varying HA concentrations, which influenced scaffold stiffness (80.98 to 190.51 kPa). PEG/SF/HA containing 50 mg HA cultured with rat BMMSCs enhanced cell adhesion, viability, the expression of all the osteogenesis-related markers in vitro and promoted superior calvarial defect repair in vivo, through modulating gene and protein expression levels [185].

Additionally, human MSCs seeded on regularly and randomly patterned photodegradable PEG hydrogel surfaces with different stiff-to-soft ratios from ~2–3 kPa to ~10–12 kPa displayed higher cell spread, elongated morphologies, and superior YAP activation and osteogenic differentiation on the regularly patterned regions, as compared to those cultured on random patterns [186]. High PEG substrate stiffness (~25 kPa) and α5β1 integrin signaling stimulated by c(RRETAWA) induced osteogenic differentiation of human MSCs [187].

#### 4.2.2. Polydimethylsiloxane

Polydimethylsiloxane (PDMS) is characterized by its biocompatibility, flexibility, optical clarity, and elastic tunability [188]. Dental follicle stem cells (DFCs) [189], and human exfoliated deciduous teeth (SHED) [190] were cultured on elastic PDMS substrates. Different stiffnesses, ranging from 11 to 93 kPa, were attained by changing the Sylgard^®^’s crosslinker to base ratios (1:55, 1:45, and 1:35 by weight) [189,190]. Coating PDMS with fibronectin caused a slight increase in ALP-activity of DFCs and continuous expression of cementoblast marker CP23 on standard cell culture dishes [189]. Osteogenic differentiation of SHED and DFCs was not supported by similar grades of ECM stiffness. In a study that involved adding osteogenic differentiation medium to DFCs on PDMS, DFCs revealed a significantly higher ALP activity and accumulation of calcium on the softest substrate (PDMS 1:55) [189], while SHED demonstrated high osteogenic differentiation on PDMS (1:35) stiffer substrate [190].

ASCs were cultured on soft and stiff PDMS substrates with moduli of elasticity ranging from (0.046 ± 0.02 MPa) and (1.014 ± 0.15 MPa), respectively. Stiff substrate enhanced the directed differentiation of ASCs into osteogenic lineages as evidenced by positive ALP stain. This enhancement was supplemented with the upregulated expression of Runx2 and Osx transcriptional factors [38].

Osteogenic differentiation of rat MSCs incubated in osteogenic medium grown on PDMS, with stiffness gradients that ranged from 0.19 to 3.10 MPa, utilizing a temperature gradient during curing, was proven to be strongly influenced by substrate stiffness and the ECM macromolecules pre-adsorbed onto the substrates. Calcein Blue (CB)-positive bone-nodule-like colonies were only observed on the stiff end of PDMS coated with fibronectin and gelatin, while oxygen-plasma-treated surfaces were entirely devoid of CB-positive colonies after 1 week of osteoinductive culture [191].

#### 4.2.3. Vinyl Polymers

A variety of functionalized vinyl monomers are commercially available or can be synthetically customized, rendering vinyl polymer-based hydrogels useful as structurally diverse scaffolds [181]. The osteogenic capability of 3D porous scaffolds composed of polytetrafluoroethylene (PTFE) and polyvinyl alcohol (PVA) with and without graphene oxide (GO) nanoparticles was investigated. These two scaffolds were fabricated through chemical crosslinking with small amounts of boric acids and a controlled freeze-drying method. The scaffolds exhibited randomly oriented nanofibers of 2 and 650 nm and compressive moduli of 620 and 130 kPa, respectively. Human ADSCs seeded on stiffer PTFE/PVA/GO scaffolds revealed a significant elevation in ALP activity, calcium deposition, and osteogenic related genes expression as compared to the softer scaffold without graphene oxide [192].

Cylindrical PV alcohol (PVA)/HA hydrogel prepared with a liquid nitrogen–contacting gradual freezing–thawing method to produce hydrogel with a wide range stiffness gradient (between ~20 kPa and ~200 kPa). Human BMMSCs cultured on PVA/HA hydrogel favored certain stiffness ranges to get differentiated into specific cell lineages: ~20 kPa for nerve cell, ~40 kPa for muscle cell, ~80 kPa for chondrocyte, and ~190 kPa for osteoblast [193]. Moreover, a minimal hydrogel matrix stiffness of 4.47 kPa was recognized to activate transcriptional co-activator TAZ and induce MSCs’ osteogenic differentiation [194].

#### 4.2.4. Polyesters

Polyesters are popular polymers that contain ester groups in the polymer backbones, enabling them to produce biomedical hydrogels that can undergo biodegradation [181]. Poly(ether-ester-urethane) (PEEU) containing poly (ρ-dioxanone) (PPDO) and PCL segments can be electrospun into fiber meshes. PEEU fiber meshes were tailored by varying the PPDO:PCL weight ratio, thus affecting their stiffness. Human ADSCs cultured on the stiffer fiber meshes (e.g., PEEU70) significantly demonstrated enhanced osteogenic differentiation with higher levels of osteocalcin expression and ALP activity. Moreover, higher levels of HA were detected on the stiffer fiber meshes [195].

Hydrophilic degradable porous 3D nanocomposite scaffolds composed of PCL, Poly (2-hydroxyethylmethacrylate) (PHEMA), and Apacite (apatite–calcite) nanostructures (15 and 25 wt.%) with mechanical values (E ~ 7.109 MPa and σ ~ 0.414 MPa) provided a balanced microenvironment that resulted in osteogenic induction of human BMMSCs. Von Kossa staining, calcium content, and ALP results confirmed the highest bone cells’ differentiation on PCLPHEMA/25% Apacite nanocomposites [196].

#### 4.2.5. Polyacrylamide

Polyacrylamide formed from only acrylamide subunits is nonionic. Copolymerizing it with other monomers such as 2-acrylamido-2-methylpropane sulfonate or acrylate forms anionic polyacrylamide, while cationic polyacrylamide could be synthesized upon copolymerization with dimethyl diallyl ammonium. Polyacrylamide substrate is bio-inert; thus, its surface must be conjugating with adhesive ECM proteins to allow for cell attachment [197,198]. Polyacrylamide is widely utilized in literature as a model for investigating the mechanoregulatory role of substrate stiffness combined or uncombined with other parameters in osteogenic differentiation. The stiffness of polyacrylamide hydrogels is commonly modified by altering the concentration of acrylamide monomer or bis-acrylamide crosslinker [62].

Upon seeding human MSCs on 250-Pa polyacrylamide gels coated with a mixture of collagen type 1 and fibronectin, the progression of the cells throughout the cell cycle was prohibited despite the presence of serum. Conversely, the quiescent cells reentered the cell cycle when presented on a stiff polyacrylamide substrate (7.5 kPa). Moreover, the non-proliferative cells revealed an adipogenic differentiation potential upon culturing on 250-Pa gels in adipogenic media or an osteogenic potential into osteoblasts if transferred to a stiff substrate in the presence of osteogenic media [199]. Micropatterned polyacrylamide gels were fabricated with varying stiffness (10 to 40 kPa), using PDMS stamps coated with fibronectin. MSCs cultured on protein-coated gels revealed a stiffness-dependent osteogenic markers’ expression (Runx2 and osteopontin) with a maximum expression at 30 kPa [200]. Osteogenic differentiation as revealed by Runx2 expression was upregulated significantly only on collagen I-coated gels with high stiffness (80 kPa), while myogenic differentiation, as ascertained by MyoD1 expression, occurred on all gel–protein coated matrices that had a stiffness of 9 kPa. Peak MyoD1 expression was demonstrated on gels with a modulus of 25 kPa coated with fibronectin. Polyacrylamide hydrogels prepared with variable stiffnesses, ranging from 13 to 68 kPa, through varying the concentrations of bis-acrylamide (0.1%, 0.5%, and 0.7%), showed a difference in the gel morphology. Under scanning electron microscopy, gels with low stiffness (13–16 kPa) appeared flat and non-porous. On the other hand, higher stiffness matrices (48–53 kPa and 62–68 kPa) showed multiple small porosities. Such inherent porosities of polyacrylamide hydrogels could enhance the flow of culture media and better mimic the natural cellular environment, as compared to plastic and glass substrates. Moreover, BMMSCs cultured on 62–68 kPa fibronectin-coated polyacrylamide hydrogels demonstrated a polygonal morphology and revealed an osteogenic phenotype with significantly high levels of ALP, Runx2, and osteopontin [51].

The modulatory effect of extracellular matrix type and density on the mechanotransduction of stem/progenitor cells and the correlated integrin involved in signals translocation were assessed through conjugating each of the four major cell adhesion ECM proteins (fibronectin, collagen I, collagen IV, and laminin) on polyacrylamide hydrogels with tunable stiffness (soft, 3 kPa; and stiff, 38 kPa). The results revealed that increasing ECM ligand density alone can induce YAP nuclear translocation without changing substrate stiffness with a different optimized ligand density. Using antibody-blocking techniques for αvβ3-, α5-, and α2β1-integrins revealed the involvement of αvβ3-, α5-, and α2β1-integrins with fibronectin, while α5-integrin was further associated with collagen type I and IV. On the contrary, laminin was associated with α5- and α2β1-integrins. Moreover, altering ECM type resulted in modulation of human MSC osteogenesis confirmed by quantitative real-time (qRT)-PCR for Runx2 and ALP without changing substrate stiffness [201].

The mechanotransduction role of FAK, α5/β1 integrin and Wnt-signaling pathways mediated by stiff matrices, in regulating osteogenic differentiation of human MSCs cultured on 62–68 kPa fibronectin-coated polyacrylamide hydrogels were further investigated. Throughout osteogenesis, gene and protein expressions of integrin α5/β1 were enhanced, together with the expression of signaling molecules FAK, p-ERK, p-Akt, GSK-3β, p-GSK-3β, and β-catenin. Antibody blocking of integrin α5 significantly downregulated the stiffness-induced expression of osteogenic markers (Runx2, alpha-1 type I collagen, and BGLAP) with associated downregulated expression of ERK, p-ERK, FAK, and β-catenin protein. Reciprocally, GSK-3β, p-GSK-3β, Akt, and p-Akt expressions were upregulated. The presence of the Akt inhibitor Triciribine reduced the expression of p-Akt and p-GSK-3β, whereas Akt, GSK-3β, and β-catenin were unchanged. These results emphasized the role of p-Akt in regulating the expression of p-GSK-3β on 62–68 kPa ECM during osteogenesis [78].

MSCs cultivated on polyacrylamide hydrogels with elasticity (7.0 ± 1.2 and 42.1 ± 3.2 kPa) and coated with type I collagen in osteogenic medium revealed enhanced osteogenic differentiation potential on stiff substrates, with an upregulated expression of Runx2, type I collagen, and osteocalcin genes. On stiff matrices, Western blot analysis revealed an increase in mechanotransducers involved in osteogenic differentiation ROCK, FAK, and ERK1/2, whereas their inhibition resulted in decreased osteogenic markers’ expression. Furthermore, α2-integrin was upregulated on stiff matrices during osteogenesis, and its knockdown by siRNA hindered the osteogenic phenotype through FAK, ROCK, and ERK1/2. Therefore, it could be concluded that α2-integrin is involved in osteogenesis mediated by matrix stiffness [62].

Additionally, upon culturing human MSCs on poly acrylamide-co-acrylic acid hydrogels grafted with RGDs, myogenic differentiation occurred at 13–17 kPa, while osteogenic differentiation was revealed at 45–49 kPa stiffness confirmed further with positive protein immunostaining of MyoD, as well as Osx, osteocalcin, and Runx2. Stiffer matrices grafted with BMP-2 mimetic peptide (E = 47.5 kPa) also induced osteoblast lineage commitment, having a similar effect as the ones grafted with RGDs. On the contrary, the osteogenic effect of BMP-2 mimetic peptides on MSCs was inhibited on very soft microenvironments (0.76–3.21 kPa) due to F-actin cytoskeleton reorganization that inhibited BMP-induced smad1/5/8 phosphorylation and subsequent differentiation of the cells into osteoblast-like cells [118].

Umbilical cord (UC) MSCs attained similar behavior upon being cultivated on different stiffness (13–16, 35–38, 48–53, and 62–68 kPa) polyacrylamide gels coated with fibronectin. Quantitative RT-PCR results showed that soft matrices promoted adipogenic differentiation, as evident by upregulated expressions of adipocytic markers (PPARγ and C/EBPα). On the contrary, stiff matrices (48–53 kPa) enhanced the tendency of the cells to differentiate into muscles, as demonstrated by enhanced expression of desmin and MOYG. High stiffness substrates (62–68 kPa) significantly promoted the expression of osteogenic markers, such as Runx2, collagen type I, ALP, and osteocalcin [50].

Moreover, the effect of mechanical loading and biomaterial stiffness on MSCs differentiation was investigated upon cultivating MSCs in osteogenic and adipogenic media on soft (23 ± 0.3 kPa) and stiff (111 ± 2 kPa) polyacrylamide as compared to PDMS (1.5 ± 0.07 MPa) either strained with 8% cyclic strain at 1 Hz or unstrained. Without strain, the expression of ALP was markedly higher on PDMS than on both polyacrylamide types. With 8% cyclic strain, ALP expression was upregulated in all groups, with the highest expression in soft polyacrylamide. Moreover, adipogenesis was the highest on the unstrained soft polyacrylamide, while it was significantly decreased on soft and stiff polyacrylamide when strained [202].

#### 4.2.6. Self-Assembling Peptides

Human MSCs were encapsulated within a 3D culture and grown on top of 2D culture biomimetic self-assembling peptide (SAP) hydrogel containing 1 mg/mL RGDs-functionalized peptide (KFE–RGD) at the shear moduli of 0.25, 1.25, 5, and 10 kPa. Changes in adipogenic and osteogenic gene expression were relatively modest with no visual signs of differentiation as mineral deposition. The cells maintained a fibroblast-like phenotype throughout the culture period. However, on introducing 1:1 mixed adipogenic/osteogenic induction medium, the stiff matrices of 10 kPa induced the most efficient osteogenesis, with alizarin red-stained calcium deposits [203].

#### 4.2.7. Other Polymers

Indirectly 3D-printed “stiffness memory” poly(urea-urethane) (PUU)/POSS elastomeric nano-hybrid scaffolds with thermo-responsive mechanical properties that soften at body temperature by inverse self-assembling have been developed. The initial stiffness and subsequent stiffness relaxation (>10 kPa) of the scaffolds directed the proliferation and differentiation of human BMMSCs towards the osteogenic lineages on stiffer scaffolds over 4 weeks, as measured by immunohistochemistry, histology, ELISA, and qPCR, while soft substrates (<1 kPa) promoted MSCs’ chondrogenic differentiation [204]. Table 1 lists the key studies investigating the effect of polymeric matrix stiffness on osteogenic differentiation of mesenchymal stem/progenitor cells.

## 5. Conclusions

The world of biomaterials, specifically polymers, lingers to impact the biomedicine field. Various materials are currently under investigation to produce ECM with varying stiffness for tissue engineering. Matrix stiffness regulates the MSCs’ differentiation into mature specific cells by activating transcription factors that upregulate the genes responsible for the initiation and progression of particular cell linage differentiation. Rigid matrices lead to increased MSCs spreading and improved actomyosin contractility, promoting osteogenic differentiation. This enhanced potential is accompanied by increased Runx2, β-catenin, and Smad, implying the significant impact of mechanosensing of the matrix stiffness and its role in determining cell fate. The comprehensive signaling mechanisms by which micro-environmental stiffness controls the lineage specification of MSCs are still unknown. Additional research is needed to understand the variety of potential signaling forces involved in MSCs’ osteogenic differentiation that can lead to the development of new therapeutic modalities addressing bone disorders. Moreover, we believe that further research on the implicated mechanical and physical factors, such as topographic changes and external mechanical forces affecting cell properties, including cell shape and colony size, can offer a broader understanding of cell-fate determination.

## Figures and Tables

**Table 1:** Key studies on the effect of polymeric matrix stiffness on osteogenic differentiation of mesenchymal stem/progenitor cells.

Study	Cell Source	Polymer	Modification	Modulus of Elasticity	Results
**Alginate**					
Zhang et al., 2020 [141]	hMSCs	Alginate–gelatin scaffold	3D bioprinted porous scaffolds different alginate concentration (0.8%alg and 1.8%alg) and different initial cell seeding density (1.67, 5, and 15 M cells/mL)	Soft scaffold 0.66 ± 0.08 kPa Stiff scaffold 5.4 ± 1.2 kPa	UpregulatedALP-activity-related, 3D-bone-like-tissue-related, osteoblast-related, and early osteocyte-related gene expression
Freeman and Kelly, 2017 [142]	MSCs	Alginate hydrogel	3D bioprinting matrix with varying alginate molecular weight and cross linker ratio		Osteogenic differentiation with increased ALP staining
Maia et al., 2014 [143]	hMSCs	Alginate hydrogel	3D matrix with bimodal molecular weight distribution at different polymer concentrations (1 and 2 wt.%) and RGD densities (0, 100 or 200 μM	2 wt.% hydrogels (tan ∂ ᵙ 0.2), 1 wt.% hydrogels (tan ∂ ᵙ 0.4–0.6).	1 wt.% alginate hydrogel matrices upregulated hMSCs osteogenic differentiation and expressed high levels of ALP and OCN
**Collagen**					
Xie et al., 2017 [146]	hMSCs	Collagen gel	Varying polymerization temperature 4, 21, and 37 °C.	Fiber stiffness: 1.1 to 9.3 kPa Bulk stiffness: 16.4 to 151.5 Pa	Collagen gel polymerized at 37 °C resulted in 34.1% ALP positive staining
Banks et al., 2014 [147]	ADSCs	Collagen–glycosaminoglycan (CG)	Chemical Crosslinking with EDAC and NHS Covalent immobilization of PDGF-BB and BMP-2 by benzophenone photolithography	2.85 to 5 MPa	Upregulated expression of collagen 1, ALP, and OCN with increased stiffness
Hwang et al., 2019 [148]	hMSCs	Three bilayers of collagen/alginate nano film		24 and 53 MPa	Increase in alkaline phosphatase activity
Zhou et al., 2021 [149]	hMSCs	Nano-particulate mineralized collagen glycosaminoglycan	Chemical crosslinking with EDAC and NHS	3.90 −/+ 0.36 kPa	Increase in expression of ALP, collagen 1, and Runx2
Tsimbouri et al., 2017 [150]	MSCs	Collagen gel	3D collagen gel culture on the vibrational bioreactor	~108 Pa	Increased expression of Runx2, collagen I, ALP, OPN, OCN, and BMP2.
Murphy et al., 2012 [151]	MSCs	Collagen/glycosaminoglycan	DHT and EDAC crosslinking	0.5, 1, and 1.5 kPa	Osteogenic differentiation with Runx2 expression
Chen et al., 2015 [152]	Rat MSCs	3D scaffold collagen and hydroxyapatite	Coated on decellularized cancellous bone	13.00 ± 5.55 kPa, 13.87 ± 1.51 kPa, and 37.7 ± 19.6 kPa	Highest scaffold stiffness promoted higher expressions of OPN and OC
Chen et al., 2017 [205]	Rat MSCs	Collagen and hydroxyapatite, coated on decellularized cancellous bone	3D oscillatory perfusion bioreactor system	6.74 ± 1.16 kPa- 8.82 ± 2.12 kPa- 23.61 ± 8.06 kPa	Osteogenic differentiation of MSCs
**Gelatin**					
Wan et al., 2019 [133]	PDLSCs	Gelatin	Crosslinked with variable concentrations of methacryloyl	GelMA concentrations of 10, 12, and 14 wt% stiffness 25.75 ± 1.21, 59.71 ± 8.87, and 117.82 ± 9.83 kPa, respectively	Increasing matrix stiffness increased osteogenic differentiation of PDLSCs, with upregulated expression of OCN and Runx2
He et al., 2018 [134]	BMMSCs	Gelatin 3%, 6%, and 9%.	Crosslinked with transglutaminase	9% gelatin gave rise to the highest stiffness (60.54 ± 10.45 kPa), while 3% gelatin resulted in the lowest stiffness (1.58 ± 0.42 kPa)	BMMSCs encapsulated in hydrogel with highest stiffness demonstrated the highest osteogenic differentiation
Van Nieuwenhove et al., 2017 [162]	ADSCs	Gelatin with variable degrees of methacrylation (GelMA 31%, GelMA 72%, and GelMA 95%)	Covalently bound to variable ratios of pentenoates modified starch (10 v% starch and 20 v% starch)		Increase in matrix stiffness promoted osteogenic differentiation of ADSCs
Jiang et al., 2015 [163]	BMMSCs	GelMA encapsulating alendronate	Crosslinked by PEG diacrylate	stiffness increased from 4 to 40 kPa	Increased osteogenic differentiation of BMMSCs on stiffer hydrogel with higher alendronate concentration with upregulated ALP, collagen I, OCN, and calcium deposition
Sun et al., 2014 [164]	BMMSCs	Three-dimensional porous gelatin scaffolds	Crosslinked using EDC	Crosslinked scaffold demonstrated an increase in the elastic modulus from w 0.6 to ≈ 2.5 kP without any change in the scaffold internal structure	Increased stiffness increased osteogenic differentiation evidenced by increased Runx2 and OCN in vitro and increased bone formation in vivo
**Decellularized matrix and Demineralized Bone**					
Ventre et al., 2019 [165]	Murine MSCs	Decellularized MC3T3-E1-cell-derived matrix on replica from PDMS	Genipin crosslinking	Young’s modulus increased from (0.01–0.1 kPa) to (0.1–1.5 kPa).	MSCs on stiff dCDMs, revealed significant adipogenic and osteogenic differentiation potentials
Hu et al., 2018 [166]	BMMSC	Demineralized bone matrices	Controlling the decalcification duration (1 h, 12 h, and 5 d, respectively)	High: 66.06 ± 27.83 MPa, Medium: 26.90 ± 13.16 MPa Low: 0.67 ± 0.14 MPa	Low stiffness scaffolds promoted osteogenesis in vitro. Subcutaneous implantation in a rat model and in a femoral condylar defect rabbit model revealed positive OCN and OPN expression
**Hyaluronic acid (HA)**					
Zhao et al., 2014 [174]	hBMMSCs	Thiol functionalized hyaluronic acid (HA) and thiol functionalized recombinant human gelatin	Crosslinked by poly (ethylene glycol) tetra-acrylate	0.15, 1.5, and 4 kPa	Change in cell morphologies with different stiffness. Cells cultured on the 4 kPa hydrogel revealed an enhanced expression of late osteogenic genes
Cosgrove et al., 2016 [175]	Juvenile bovine MSCs	Methacrylated HA hydrogel	Ligation of the HAVDI adhesive peptide sequence from N-cadherin domain 1 and RGD from fibronectin	5, 10, and 15 kPa	Lack of myosin IIA incorporated into focal adhesions hindered their maturation with increasing substrate stiffness and decreased osteogenesis
Dorcemus et al., 2017 [176]	hMSCs-bone-marrow-derived	Thiol-modified hyaluronan gel	Crosslinked by PEG at ratios ranging from 1:1 to 7:1	Storage moduli from 10 to 45 Pa	Differences between the top (cartilage-forming) and bottom (bone-forming) regions of the scaffold proved its capability for osteochondral engineering
Hao et al., 2018 [177]	hMSCs-bone-marrow-derived	HA carrying sulfhydryl groups and a hydrophilic polymer bearing both acrylate and tetrazine groups	Matrix metalloprotease -degradable peptidic crosslinker and adding HA conjugated with multiple copies of trans-cyclooctene (TCO)	(G’) = 180 ± 42 Pa increased to G′ = 520 ± 80 Pa	The 3D matrix tagged with a TCO- motif promoted the cells to undergo change from a rounded to spindle phenotype
**Fibrin**					
Hashemzadeh et al., 2019 [180]	hADSCs	Fibrin hydrogels embedding gold nanowires	Altering fibrinogen and thrombin concentration and incorporation of gold nanowires		With high fibrinogen and thrombin concentration, gold nanowires, promoted osteogenic differentiation
**Polyethylene glycol (PEG)**					
Pek et al., 2010 [182]	MSCs	Thixotropic polyethylene glycol–silica (PEG–silica) nano composite gel	3D cell culture Cell-adhesion peptide RGD (Arg–Gly–Asp) sequence immobilization	≥75 Pa	Higher expression of the osteogenic transcription factor
Ye et al., 2015 [183]	Rat BMMSCs	PEG	PEG hydrogels with RGD nano-spacings of 49 and 135 nm and incubated in mixed osteogenic and adipogenic medium	Soft hydrogels (130 kPa) and stiff hydrogels (3170 kPa)	Stiff hydrogels promoted osteogenesis. Large RGD nano-spacing promoted osteogenesis
Steinmetz et al., 2015 [184]	hMSCs	Multilayer PEG-based hydrogel	Simple sequential photopolymerization- high RGD concentrations- dynamic mechanical stimulation	345 kPa	Collagen I generation with mineral deposits were evident
Yang et al., 2020 [185]	Rat BMMSCs	PEG/silk fibroin/HA scaffold	Varying HA concentration	80.98 to 190.51 kPa	Expression of all the osteogenesis-related markers in vitro and superior calvarial defect repair in vivo
Yang et al., 2016 [186]	hMSCs	PEG hydrogel	Regularly and randomly patterned photodegradable hydrogel	∼10–12 kPa	Osteogenic differentiation of MSCs cultured on random patterns
Gandavarapu et al., 2014 [187]	hMSCs	PEG hydrogels	functionalized with c(RRETAWA) hydrogels through α5 integrins	∼25 kPa	Osteogenic differentiation of hMSCs
**Polydimethylsiloxane (PDMS)**					
Xie et al., 2018 [38]	ASCs	PDMS		1.014 ± 0.15 MPa	Osteogenic differentiation by ALP stain and upregulation of Runx2 and Osx transcriptional factors
Viale-Bouroncle et al., 2014 [189]	DFCs	PDMS	Coating PDMS with fibronectin and cultured in osteogenic differentiation medium	11 kPa	High ALP activity and accumulation of calcium on the soft substrate
Viale-Bouroncle et al., 2012 [190]	SHED	PDMS	Adding osteogenic differentiation medium	93 kPa	High osteogenic differentiation
Wang et al., 2012 [191]	Rat MSCs	PDMS	Osteogenic medium with temperature gradient curing	0.19 to 3.10 MPa	Calcein Blue–positive bone-nodule-like colonies
**Vinyl polymers**					
Khoramgah et al., 2020 [192]	hADSCs	Poly tetra fluoro ethylene (PTFE) and PVA with and without graphene oxide nanoparticles	3D porous scaffolds- chemical crosslinking with small amounts of boric acids–controlled freeze-drying method	620 and 130 kPa	Elevation in ALP activity, calcium deposition, and osteogenic-related genes expression
Oh et al., 2016 [193]	hBMMSCs	Cylindrical PVA/HA hydrogel	Liquid nitrogen—contacting gradual freezing–thawing method	~20 kPa and ~200 kPa	Stiffness of ~190 kPa led to osteoblast differentiation
**Polyesters**					
Sun et al., 2019 [195]	hADSCs	Poly(ether-ester-urethane) (PEEU) containing PPDO and PCL segments	Electrospun into fiber meshes with varying PPDO to PCL weight ratios	2.6 ± 0.8 MPa (PEEU40), 3.2 ± 0.9 MPa (PEEU50), 4.0 ± 0.9 MPa (PEEU60) 4.5 ± 0.8 MPa (PEEU70)	Enhanced osteogenic differentiation of hADSCs with higher levels of OCN, ALP, and hydroxyapatite detected on the stiffer fiber meshes
**Self-assembling peptides**					
Hogrebe and Gooch, 2016 [203]	hMSCs	Biomimetic self-assembling peptide hydrogel containing 1 mg/mL RGD-functionalized peptide (KFE–RGD)	hMSCs were encapsulated within 3D culture and grown on top of 2D culture Adding 1:1 mixed adipogenic/osteogenic induction medium	(G′) 10 kPa	Osteogenesis induction and alizarin red-stained calcium deposits
**Other Polymers**					
Olivares-Navarrete et al., 2017 [76]	MSCs	Methyl acrylate/methyl methacrylate polymer	Altering monomer concentration.	0.1 MPa to 310 MPa	Chondrogenic and osteogenic differentiation when grown on substrates with less than 10 MPa stiffness
Wu et al., 2018 [204]	hBMMSCs	Poly(urea-urethane) (PUU)/POSS elastomeric nano-hybrid scaffolds	Thermoresponsive scaffolds indirectly 3D printed by inverse self-assembling	>10 kPa	Osteogenic differentiation

## Data Availability

Not applicable.

## Abbreviations

3D	Three-dimensional
ADSCs	Adipose derived stromal cells
ALP	Alkaline phosphatase
ASCs	adipose derived stromal cells
BMMSCs	Bone marrow mesenchymal stem cells
BMP-2	Bone morphogenetic protein 2
dCDMs	Decellularized cell derived matrix
DFCs	Dental follicle stem cells
ECM	Extracellular matrix
EDAC	1-ethyl-3-(3-dimethylami-nopropyl) carbodiimide
GC-TRS	Glycol chitin-based thermo-responsive hydrogel scaffold
GelMA	Gelatin with variable degrees of methacrylation
HA	Hyaluronic acid
HA	Hydroxyapatite
hADSCs	Human adipose derived mesenchymal stem cells
hBMMSCs	Human Bone marrow mesenchymal stem cells
hMSCs	Human mesenchymal stem cells
MSCs	Mesenchymal stem cells
NHS	N-hydroxysuccinimide
OCN	Osteocalcin
ON	Osteonectin
OPN	osteopontin
PCL	poly (ε-caprolactone)
PDLSCs	periodontal ligament stem cells
PDMS	Polydimethylsiloxane
PEEU	Poly (ether-ester-urethane)
PEG	Polyethylene glycol
PEGDA	Polyethylene glycol diacrylate
PEGMC	Polyethylene glycol-maleate-citrate
PPDOP	poly (*ρ*-dioxanone)
PV	polyvinyl
PVA	polyvinyl alcohol
qRT-PCR	Quantitative Reverse transcription real-time polymerase chain reaction
RGD	Arginine-glycine-aspartic acid
Runx2	Runt-related transcription factor-2
SCAP	Stem cells of the apical papilla
SDF-1α	Stromal derived factor-1alpha
SHED	Stem cells isolated from human exfoliated deciduous teeth
